# The Inhibitory Effect of the Hepatitis B Virus Singly-Spliced RNA-Encoded p21.5 Protein on HBV Nucleocapsid Formation

**DOI:** 10.1371/journal.pone.0119625

**Published:** 2015-03-18

**Authors:** Yi-Ling Wang, Gan-Guang Liou, Chao-Hsiung Lin, Mong-Liang Chen, Tzer-Min Kuo, Kuen-Nan Tsai, Chien-Choao Huang, Ya-Ling Chen, Li-Rung Huang, Yu-Chi Chou, Chungming Chang

**Affiliations:** 1 Institute of Microbiology and Immunology, National Yang-Ming University, Taipei, Taiwan; 2 Institute of Molecular and Genomic Medicine, National Health Research Institutes, Miaoli, Taiwan; 3 Institute of Biomedical Informatics, National Yang-Ming University, Taipei, Taiwan; 4 Center for Molecular Medicine, China Medical University and Hospital, Taichung, Taiwan; 5 Institute of Molecular Biology, Academia Sinica, Taipei, Taiwan; Academia Sinica, TAIWAN

## Abstract

Hepatitis B virus (HBV) is the smallest DNA virus and the major cause of acute and chronic hepatitis. The 3.2 kb HBV viral genome generates four major species of unspliced viral transcript as well as several alternatively spliced RNAs. A 2.2 kb singly-spliced RNA is the most abundant spliced RNA and is widely expressed among all HBV genotypes. The expression of the singly-spliced RNA, as well as that of its encoded protein HBSP, is strongly associated with hepatopathology during HBV infection. Here, we report a novel inhibitory role of a p21.5 protein, which is encoded by a 2.2 kb singly-spliced RNA, in the modulation of HBV replication. We show that overexpression of the singly-spliced RNA is able to efficiently inhibit HBV replication. Furthermore, a mutation in the ATG start codon of the precore region completely abolishes the inhibitory effect of the singly-spliced RNA, indicating that a viral protein (p21.5) derived from the singly-spliced RNA is the mediator of the inhibition. Furthermore, p21.5 is able to form a homodimer that interacts with core dimers forming hybrid viral assembly components. Sucrose gradient fractionation revealed that co-expression of p21.5 resulted in a spread distribution pattern of core proteins ranging from low to high sucrose densities. When compared with p22, p21.5 is almost ten times more efficient at destabilizing HBV nucleocapsid assembly in Huh7 cells overexpressing either p21.5 or p22 protein. Moreover, *in vivo* expression of p21.5 protein by tail vein injection was found to decrease the amount of nucleocapsid in the livers of HBV-expressing BALB/c mice. In conclusion, our study reveals that the HBV 2.2 kb singly-spliced RNA encodes a 21.5 kDa viral protein that significantly interferes with the assembly of nucleocapsids during HBV nucleocapsid formation. These findings provide a possible strategy for elimination of HBV particles inside cells.

## Introduction

Hepatitis B virus (HBV), a member of the hepadnaviridae family, is the smallest DNA virus and the major cause of acute and chronic hepatitis. More than 350 million people worldwide are living with chronic hepatitis B (CHB), which causes progressive liver damage and leads to the development of cirrhosis and hepatocellular carcinoma (HCC) [1, 2]. The 3.2 kb HBV genome transcribes four major species of unspliced RNA transcripts that are essential for HBV replication [3, 4], as well as a variety of spliced viral RNAs [5]. However, only a few of the spliced RNAs have been well studied and most of their biological functions remain unclear.

In HBV-producing hepatoma cells, a 2.2 kb singly-spliced RNA that lacks intron 2447/489 is the most abundant spliced viral RNA and represents between 20% and 30% of the spliced viral transcripts [6, 7]. Although initial analysis has suggested that this singly-spliced RNA is not essential for HBV replication [7], further studies have suggested that the existence of this singly-spliced RNA is closely related to hepatopathology during HBV infection. The singly-spliced RNA has been shown to able to be encapsidated into capsids to form defective viral particles both *in vivo* and *in vitro* [5, 7–11]; furthermore, it can be detected in the sera of 95% of CHB patients [9]. High levels of defective viral particles have been observed in acute hepatitis patients who progress to chronic hepatitis, suggesting that the singly-spliced RNA is closely associated with viral persistent infection [9]. Moreover, an elevated proportion of defective viral particles to total viral particles has been shown to be highly correlated with severe hepatopathologies, including necrosis and fibrosis [12].

The 2.2 kb singly-spliced RNA has been reported to encode an HBV splice-generated protein (HBSP) [13]. Several studies have been conducted to elucidate the biological functions of the HBSP during HBV-mediated pathogenesis. HBSP has been detected in liver biopsy samples obtained from patients with HBV infection [13], and the level of HBSP has been found to be associated with the severity of liver fibrosis in CHB patients [14]. It has also been reported that HBSP activates HBSP-specific T cell responses in the peripheral blood mononuclear cells of CHB patients [15]. Ectopic expression of HBSP was found to cause cell apoptosis in transfected hepatoma cells [13, 16]. Although the existence of this singly-spliced RNA has been known for decades, HBSP is the only functional viral protein produced from this RNA that has had its functions explored so far. However, the singly-spliced RNA also contains another open reading frame; this encodes a precore/core protein that is a one-amino acid shorter and up to the present the functional roles of this splice-generated protein has not been studied.

In this study, we report that a splice-generated protein (p21.5), derived from the one-amino acid shorter precore/core protein, exerts an inhibitory effect on HBV capsid formation. Overexpression of the 2.2 kb singly-spliced RNA was found to increase the expression level of p21.5 and to reduce significantly the amount of nucleocapsids in HBV-expressing hepatoma cells. In addition, p21.5 was found to form a homodimer that interacts with the core dimer; this interaction interferes with nucleocapsid formation both *in vitro* and *in vivo*. The inhibitory effect by which this singly-spliced RNA modulates HBV capsid formation is explored in the present study.

## Materials and Methods

### Ethics statement

This study was conducted under the approval of Institutional Animal Care and Use Committee (IACUC) of National Health Research Institutes (Protocol No. NHRI-IACUC-102002-A).

### Cell culture

The human hepatoma cell lines HepG2 and Huh7, and human embryonic kidney 293T cells were maintained in Dulbecco’s modified Eagle's medium (DMEM) (Gibco Laboratories, Grand Island, NY) supplemented with 10% fetal bovine serum (FBS), 2 mM L-glutamine, and 100 μM non-essential amino acids. They were grown at 37°C in 5% CO_2_, and 100% relative humidity in an incubator.

### Plasmid constructs and DNA transfection

p1.3HBcl/Hyg is an HBV-expressing plasmid that contains 1.3 copies of the entire HBV genome and is able to support HBV replication [17]. pShuttle/R vector was obtained from the Adeno-X Expression System (Clontech). pSh3100HBcl was constructed by cloning a fragment containing a single copy HBV genome into the pShuttle/R vector. pShPC/R was constructed by cloning the cDNA sequence of the HBV precore open reading frame (ORF) into the pShuttle/R vector. pShCore/R was constructed by cloning the cDNA sequence of the HBV core open reading frame (ORF) into the pShuttle/R vector. pShSS/R was constructed by cloning a fragment of the cDNA sequence of singly-spliced RNA (nt 1814–2447/489–1835, numbering starting at the EcoRI site) into the pShuttle/R vector. pShSScore^-^/R was constructed by cloning a fragment of the cDNA sequence of singly-spliced RNA with a single mutant of nucleotide A to T (nt 1919) into the pShuttle/R vector. This mutant is able to express singly-spliced RNAs but produces core protein that is only 6 amino acids in length. pShSS_prestop mutant/R was constructed by cloning a fragment of the cDNA sequence of singly-spliced RNA with a single mutant of nucleotide C to T (nt 2428, numbering starting at the EcoRI site) into the pShuttle/R vector to generate a prestop codon at the 7^th^ amino acid upstream of the authentic stop codon of p21.5. pShPC-1/R was constructed by cloning the sequence of the precore/core ORF of the singly-spliced RNA into the pShuttle/R vector. pShSS_PCmut/R and pShSS_Cmut/R were derived from pShPC-1/R by mutating the ATG start codon of the precore ORF and core ORF to GTG. pShSSFlag/R was also derived from pShPC-1/R and had amino acids 78–81 (DPAS) of the core ORF replaced by the FLAG sequence (DYKDDDDK) as previously described [18]. pSh22/R was constructed by cloning the coding sequence of the HBV p22 protein (nt 1871–2449, numbering starting at the EcoRI site) with an artificial ATG into the pShuttle/R vector as previously described [18].

DNA transfection was performed using jetPEI polymer-based DNA transfection reagent (Polypus-Transfection). In brief, 3×10^6^ cells were plated in 10 ml of the Dulbecco’s modified Eagle's medium (DMEM) (Gibco Laboratories, Grand Island, NY) supplemented with 10% fetal bovine serum (FBS) per 10-cm dish one day before transfection. After culturing overnight, the medium was replaced with 5 ml of 10% DMEM medium before transfection. For each dish, 10 μg of DNA was diluted into 250 μl 150 mM NaCl. In parallel, 20 μl of jetPEI reagent was diluted into 250 μl 150 mM NaCl. The diluted jetPEI reagent was combined with the diluted DNA and incubated for 15 minutes at room temperature. The DNA-jetPEI complexes were then added to the dishes and mixed gently by rocking the plate back and forth. Finally, the dishes were incubated at 37°C in a CO_2_ incubator.

### Generation of recombinant adenoviruses and infection of hepatoma cell lines

Recombinant adenoviruses (AdIE, AdSS, and Ad1.3HBV) were prepared according to the BD Adeno-X Expression System 1 User Manual (Clontech). In brief, a gene fragment was cloned from the pShuttle vector into the pAdeno-X vector, and pAdeno-X was transfected into HEK-293T cells. The recombinant adenoviruses carrying the EGFP (AdIE) gene, singly-spliced RNA (AdSS), or 1.3 copies HBV (Ad1.3HBV) were collected from the cells. To infect HepG2 cells with adenovirus, cells were plated on a 10 cm-dish 24 h before infection. The next day, the growth medium was removed and 5 ml of virus (diluted to achieve the desired MOI) was then added to the dish. The plates were tipped to spread the viruses evenly and the dish was incubated at 37°C in a CO_2_ incubator to allow the virus to infect the cells. After 2 hours, 5 ml fresh complete growth medium was added. The cells were then incubated at 37°C for 3 or 5 days.

### Extraction and analysis of viral RNA

For HBV RNA analysis, total RNA was extracted following the TRIzol RNA Isolation Reagent (Invitrogen) protocol. Equal amounts of total RNA were then separated by gel electrophoresis and analyzed by northern blot hybridization using a ^32^P-labeled HBc DNA fragment.

### Detection of viral proteins

Cells were lysed in NET buffer (50 mM Tris-Cl, 125 mM NaCl, 1 mM EDTA, 0.5% Nonidet P-40, pH7.5) in the presence of protease inhibitor cocktails. Equal amounts of total proteins were then separated by SDS-polyacrylamide gel electrophoresis (SDS-PAGE) and transferred onto polyvinylidene fluoride membrane (Millipore, Billerica, MA). Each membrane was then blocked with 5% nonfat milk in 1× PBS, which was followed by incubation with anti-HBV core antibody (mouse monoclonal antibody prepared by Dr. C. Hu, which is specific against the epitope of HBV precore/core protein, a.a. -3–9 and 75–86, numbering starting at the first amino acid of core O.R.F.), anti-GFP antibody (Clontech), or anti-beta-actin antibody (Sigma). The immunoblot signals were examined using enhanced chemiluminescence reagent (PerkinElmer Life Sciences).

### Particle blot analysis

Analysis of intracellular HBV core particles was performed as previously described [19]. In brief, equal amounts of total protein were separated using a 1.2% native agarose gel and then transferred onto polyvinylidene fluoride membrane. The presence of core particles was examined by immunoblot analysis using anti-core antibody (DakoCytomation). To detect capsid-associated nucleic acids, the samples were transferred onto nylon membranes. Capsid-associated nucleic acids were released from the core particles *in situ* by denaturing the membranes with 0.2 N NaOH/1.5 M NaCl followed by neutralizing with 0.2 N Tris-HCl/1.5 M NaCl. Finally, the membranes were hybridized with ^32^P-labeled HBx DNA fragment.

### Viral nucleocapsid isolation

Equal amounts of total proteins were ultracentrifuged at 500,000 × g through 2 ml of a 20% sucrose–NET (50 mM Tris-Cl, 125 mM NaCl, 1 mM EDTA, 0.5% Nonidet P-40, pH7.5) (wt/wt) cushion for 1 h at 4°C using a TLA 100 rotor (Beckman Instruments, Palo Alto, CA). Under these conditions, viral core particles are pelleted, whereas free core protein and soluble HBeAg remain in the supernatant as has been described previously [18]. The pelleted material was resuspended in NET (50 mM Tris-Cl, 125 mM NaCl, 1 mM EDTA, 0.5% Nonidet P-40, pH7.5) buffer and analyzed by Southern blot or western blot analysis.

### Viral DNA extraction and analysis

HBV replication following transient transfection of cell lines was assessed by Southern blot analysis as described previously with some modifications [18]. The isolated viral nucleocapsids were treated with 50 μg/ml proteinase K at 52°C for 1 hour. Viral DNA was purified by phenol/chloroform extraction and separated on a 1.2% agarose gel. After electrophoresis, the gel was soaked in denaturing buffer (0.5 M NaOH, 1.5 M NaCl) twice for 15 min each time and then neutralized with neutralizing buffer (1 M Tris-HCl [pH8.0], 1.5 M NaCl). The HBV DNA that was released was then transferred onto a nylon membrane and detected by hybridization with a ^32^P-labeled HBx-specific probe.

### Sucrose gradient analysis

Cells were lysed in NET buffer (50 mM Tris-Cl, 125 mM NaCl, 1 mM EDTA, 0.5% Nonidet P-40, pH7.5) in the presence of protease inhibitor cocktail. Equal amounts of total proteins were then layered onto 10 ml of a 10 to 60% (wt/wt) sucrose–NET (50 mM Tris-Cl, 125 mM NaCl, 1 mM EDTA, 0.5% Nonidet P-40, pH7.5) mixture. Gradients were established by ultracentrifugation at 30,000 rpm with a SW41 rotor for 17 h at 4°C. Fourteen 0.75-ml aliquots were collected from the gradient and equal volumes were then analyzed by western blot to detect the proteins that were present.

### Animal study

BALB/c mice (male, 6–8 weeks old, from the National Laboratory Animal Center, Taiwan) were anesthetized with isoflurane. Fifteen micrograms of plasmid DNA was injected into the tail vein of each mouse in a volume of PBS equivalent to 8% of the mouse body weight. The total volume was delivered within 5 seconds. Liver samples were then collected for particle blot analysis on day 3 after injection.

### Quantification of HBsAg

The HBsAg in mice serum was measured with an enzyme-linked immunosorbent assay (ELISA) Kit purchased from General Biology Corp. The amount of formazan dye formed correlated with the amount of HBsAg, which was determined quantitatively using a scanning multi-well spectrophotometer (ELISA reader) at an absorbance of 450 nm. By using standards of HBsAg with known concentrations, the amounts of the secreted HBsAg were estimated.

## Results

### HBV replication is inhibited by overexpression of the singly-spliced 2.2 Kb RNA

**Fig 1:**
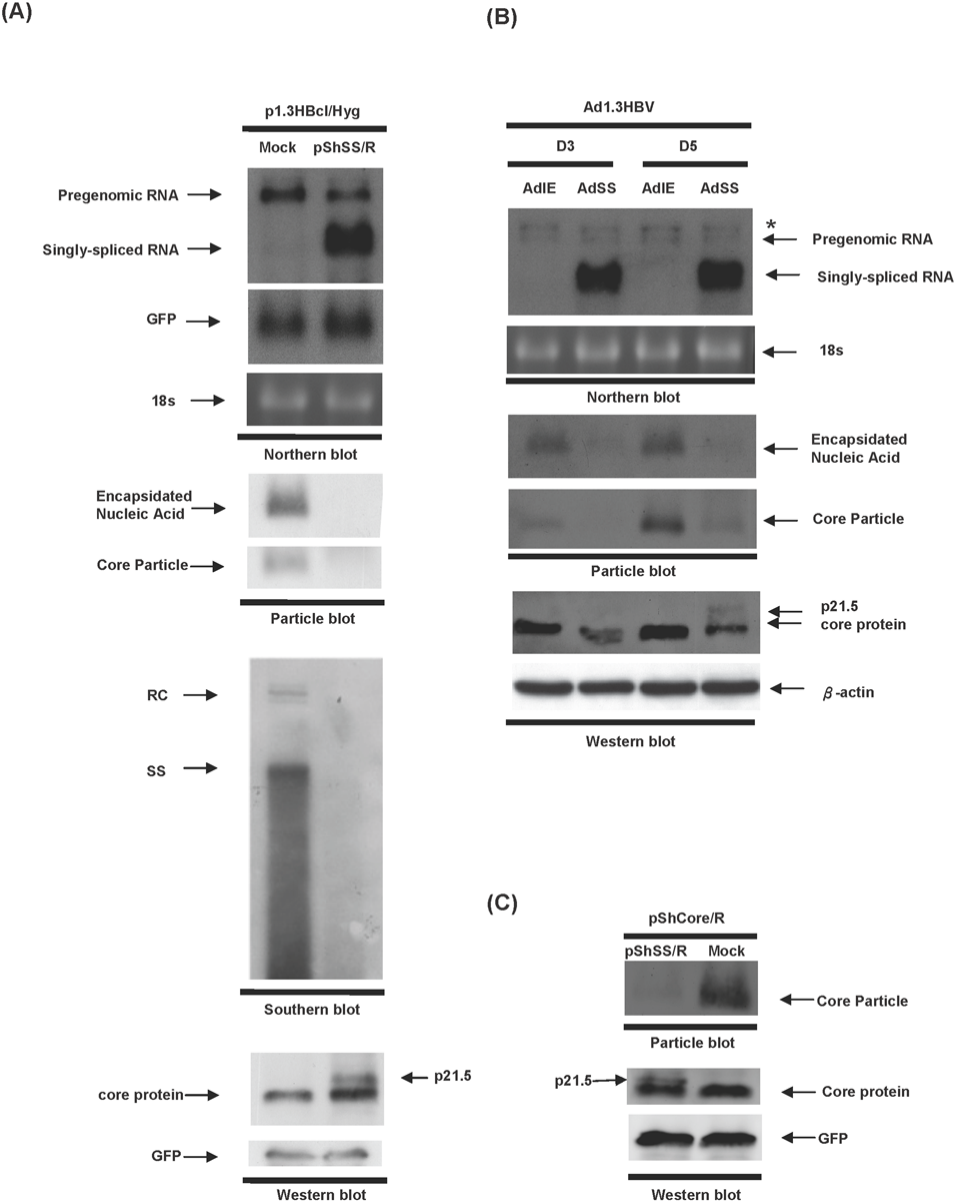
HBV replication is inhibited by overexpression of singly-spliced RNA. (A) Huh7 cells were co-transfected with p1.3HBcl/Hyg together with either pShSS/R or control plasmid (pShuttle/R, Mock) and the cells were harvested three days post-transfection. Top panel, the expression levels of pgRNA transcript and singly-spliced RNA were revealed by northern blot analysis using hybridization with an HBc-specific probe. The expression level of co-transfected GFP was used as a transfection control and 18s rRNA was used as a loading control. Second panel, the formation of intracellular nucleocapsids was detected by particle blot analysis. Third panel, HBV DNA extracted from intracellular core particles was analyzed by Southern blot analysis. Bottom panel, the expression level of HBV core protein was analyzed by western blot analysis. The expression of GFP protein was used as a loading control. (B) HepG2 cells were co-transduced with Ad1.3HBV together with either AdSS or AdIE for 3 days or 5 days. Total RNA was extracted from the transduced cells and analyzed by northern blot analysis using an HBc-specific probe. The star mark indicates the 3.9 kb X-mRNA generated from the 1.3 copies of the HBV genome of Ad1.3HBV [20]. Total proteins were harvested for particle blot and western blot analyses. The encapsidated nucleic acid was revealed by hybridizing with an HBx-specific probe. The expression level of core protein was detected using anti-core antibody. (C) Huh7 cells were co-transfected with pShCore/R together with either pShSS/R or a control plasmid (Mock), and cell lysate was harvested for particle blot and western blot analyses using anti-core antibody at day 3 post-transfection.

In order to investigate the effect of the singly-spliced 2.2 kb RNA on HBV replication, Huh7 cells were cotransfected with plasmids expressing HBV (p1.3HBcl/Hyg) together with the singly-spliced RNA (pShSS/R) or a control plasmid pShuttle/R (Mock); then the replication capacities of HBV in the presence or absence of singly-spliced RNA expression were analyzed. A dramatic decrease in the amount of nucleocapsid was observed when there was overexpression of the singly-spliced RNA (second panel, Fig. 1A, particle blot). Southern blot analysis also demonstrated that HBV replication was significantly suppressed by overexpression of the singly-spliced RNA (third panel, Fig. 1A, Southern blot). However, the expression level of HBV pregenomic RNA (pgRNA) was only slightly decreased by overexpression of the singly-spliced RNA (top panel, Fig. 1A, northern blot). Interestingly, a novel 21.5 kDa viral protein (p21.5) with lower electrophoretic mobility than the wild-type core protein was detected when the singly-spliced RNA was overexpressed (lower panel, Fig. 1A, western blot).

We next examined the inhibitory effect of singly-spliced RNA in other hepatoma cells using recombinant adenovirus expressing HBV (Ad1.3HBV) together with expression of either EGFP (AdIE) or singly-spliced RNA (AdSS); these were co-transduced into HepG2 cells. A similar inhibitory effect of the singly-spliced RNA on HBV replication was observed in HepG2 cells. Furthermore, while the expression level of pgRNA was also slightly reduced after adenovirus AdSS transduction (top panel, Fig. 1B, northern blot), interestingly the level of intracellular nucleocapsids was significantly reduced by the expression of the singly-spliced RNA (middle panel, Fig. 1B, particle blot). Slowly-migrating p21.5 was also detected in the AdSS-transduced HepG2 cells (lower panel, Fig. 1B, western blot). It should be noted that a very small amount of p21.5 (less than 10% of the wild-type core protein) was sufficient to efficiently inhibit intracellular nucleocapsid formation (Ad1.3HBV and AdSS, D5, Fig. 1B, particle blot and western blot). Since HBV core proteins can spontaneously self-assemble into viral capsids in the absence of pgRNA, we next examined whether p21.5 exerts its inhibitory effect by directly interfering with the self-assembly of core proteins (Fig. 1C). Huh7 cells were co-transfected with plasmids expressing HBV core protein (pShCore/R) together with either singly-spliced RNA (pShSS/R) or control plasmid (Mock). The cell lysates were then harvested and subjected to northern blotting, particle blotting, and western blotting. The expression of core mRNA was detected by northern blot analysis using hybridization with an HBc-specific probe and shown in S1 Fig. It was found that the amount of assembled core particles was dramatically decreased by overexpression of the singly-spliced RNA, while at the same time the expression level of HBV core proteins was not significantly changed (Fig. 1C). Taken together, these experiments demonstrate that overexpression of the singly-spliced RNA significantly decreases the amount of HBV nucleocapsids produced in various different hepatoma cell lines, probably by interfering with viral capsid formation.

### Characterization of the 21.5 kDa viral protein encoded by the singly-spliced RNA

**Fig 2:**
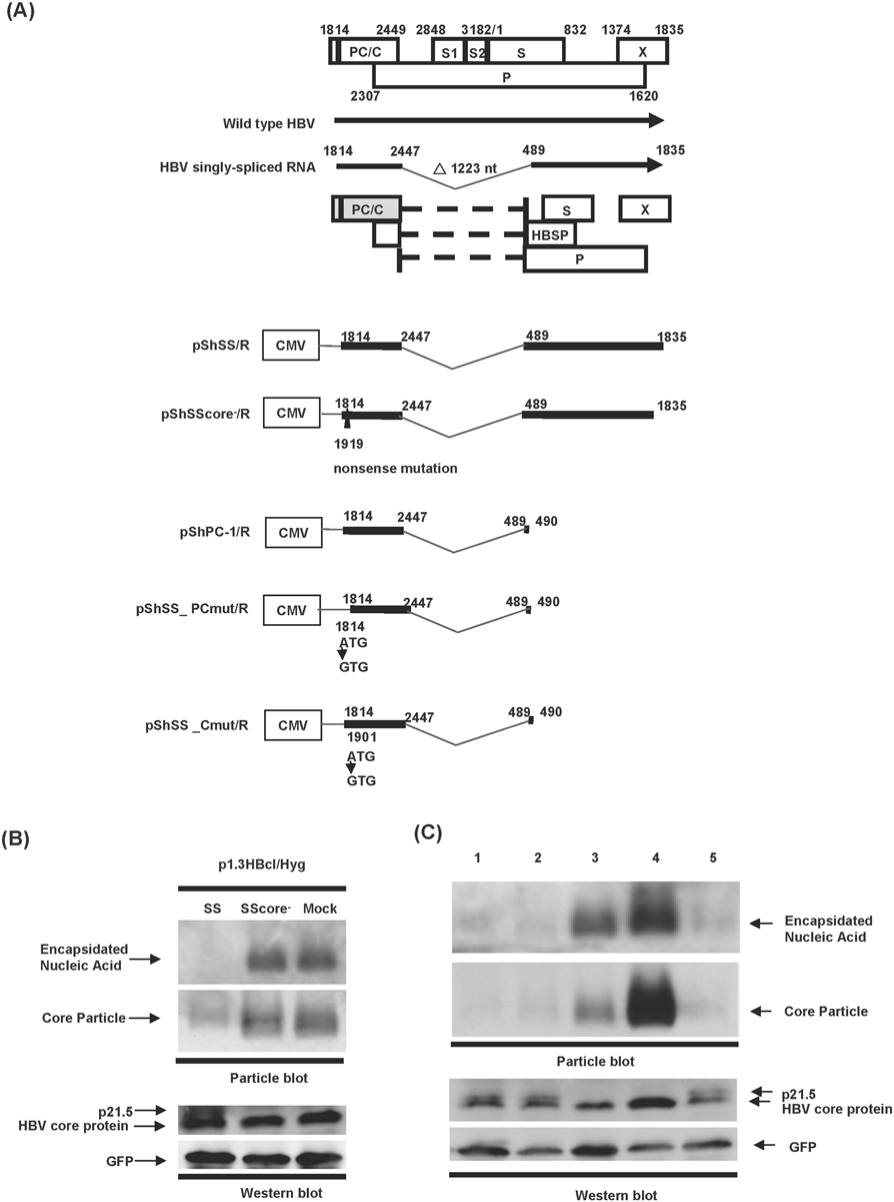
The singly-spliced RNA encodes a 21.5 kD viral protein that inhibits HBV replication. (A) Top panel, schematic of the structure and open reading frames of the singly-spliced RNA. Lower panel, schematic of the plasmid constructs. pShSS/R, a plasmid expressing full-length singly-spliced RNA. pShSScore^-^/R, a plasmid expressing singly-spliced RNA with a nonsense mutation within the precore/core ORF. pShPC-1/R, a plasmid expressing singly-splicedRNA with 3' UTR truncation. pShSS_PCmut/R, a plasmid derived from pShPC-1/R in which the ATG of precore ORF was mutated to GTG. pShSS_Cmut/R, a plasmid derived from pShPC-1 in which the ATG of core ORF was mutated to GTG. (B) and (C) Huh7 cells were transfected with the indicated plasmids for three days, and total cell lysate was harvested. In the top two panels, the expression levels of nucleocapsid were analyzed by particle blot analysis. The encapsidated nucleic acid was revealed by hybridizing with an HBx-specific probe. In the lower two panels, the expression levels of HBV core protein and GFP protein were examined by western blot analysis. (C) Lane 1: p1.3HBcl/Hyg and pShSS/R; lane 2: p1.3HBcl/Hyg and pShPC-1/R; lane 3: p1.3HBcl/Hyg and pShShuttle/R (Mock); lane 4: p1.3HBcl/Hyg and pShSS_PCmut/R; lane 5: p1.3HBcl/Hyg and pShSS_Cmut/R.

The detection of a 21.5 kDa viral protein (p21.5) in hepatoma cells that are overexpressing singly-spliced RNA raised the possibility that this viral protein may act as a mediator in the inhibition of nucleocapsid assembly. To investigate this possibility, a nonsense mutation was made within the plasmid pShSS/R by introducing a premature stop codon at nt 1919 (numbering starting at the EcoRI site) in order to terminate the translation of both the precore and core proteins (Fig. 2A, pShSScore^-^/R). Interestingly, the formation of HBV capsids was inhibited by cotransfection with pShSS/R but not with pShSScore^-^/R (Fig. 2B), which indicates that a precore/core-derived protein encoded by this singly-spliced RNA is responsible for its inhibitory effect on nucleocapsid formation. To determine whether the precore/core ORF alone is sufficient to exert an inhibitory effect, a region containing the putative 3'UTR (nt 491–1835) of the singly-spliced RNA was removed (Fig. 2A, pShPC-1/R). Importantly, pShPC-1/R is able to generate a 3'UTR-truncated singly-spliced RNA that has an inhibitory effect on nucleocapsid formation equivalent to intact singly-spliced RNA (Fig. 2C, lane 1 and lane 2). This result confirms that the coding region of the singly-spliced RNA-encoded viral protein is located within the precore/core ORF. Considering the gene structure of the singly-spliced RNA, the coding region of this novel viral protein is likely to begin at either the precore ATG or the core ATG and to terminate at the end of the core ORF right before the authentic core termination codon (Fig. 2A). To explore the exact translation start site of this novel viral protein, the ATG at the translation start site of either the precore or core ORF of the singly-spliced RNA was mutated to GTG (Fig. 2A, pShSS_PCmut/R and pShSS_Cmut/R, respectively). As shown in Fig. 2C, a mutation at the ATG codon of the precore completely abolished the inhibition of nucleocapsid formation, as well as the generation of p21.5 (Fig. 2C, lane 4). The unexpected higher level of nucleocapsids on cotransfection with pShSS_PCmut/R is likely to be due to the increased amount of core protein translated from pShSS_PCmut/R (Fig. 2C, lane 4). On the other hand, an ATG mutation at the core start site did not affect the inhibitory effect of the singly-spliced RNA (Fig. 2C, lane 5). These results reveal that the functional execution of the singly-spliced RNA occurs via the 21.5 kDa viral protein (p21.5) which is translated from the precore ATG and contains a precore/core coding sequence.

### The 21.5 kDa viral protein forms homodimers that interact with core protein dimers and form a hybrid structure

**Fig 3:**
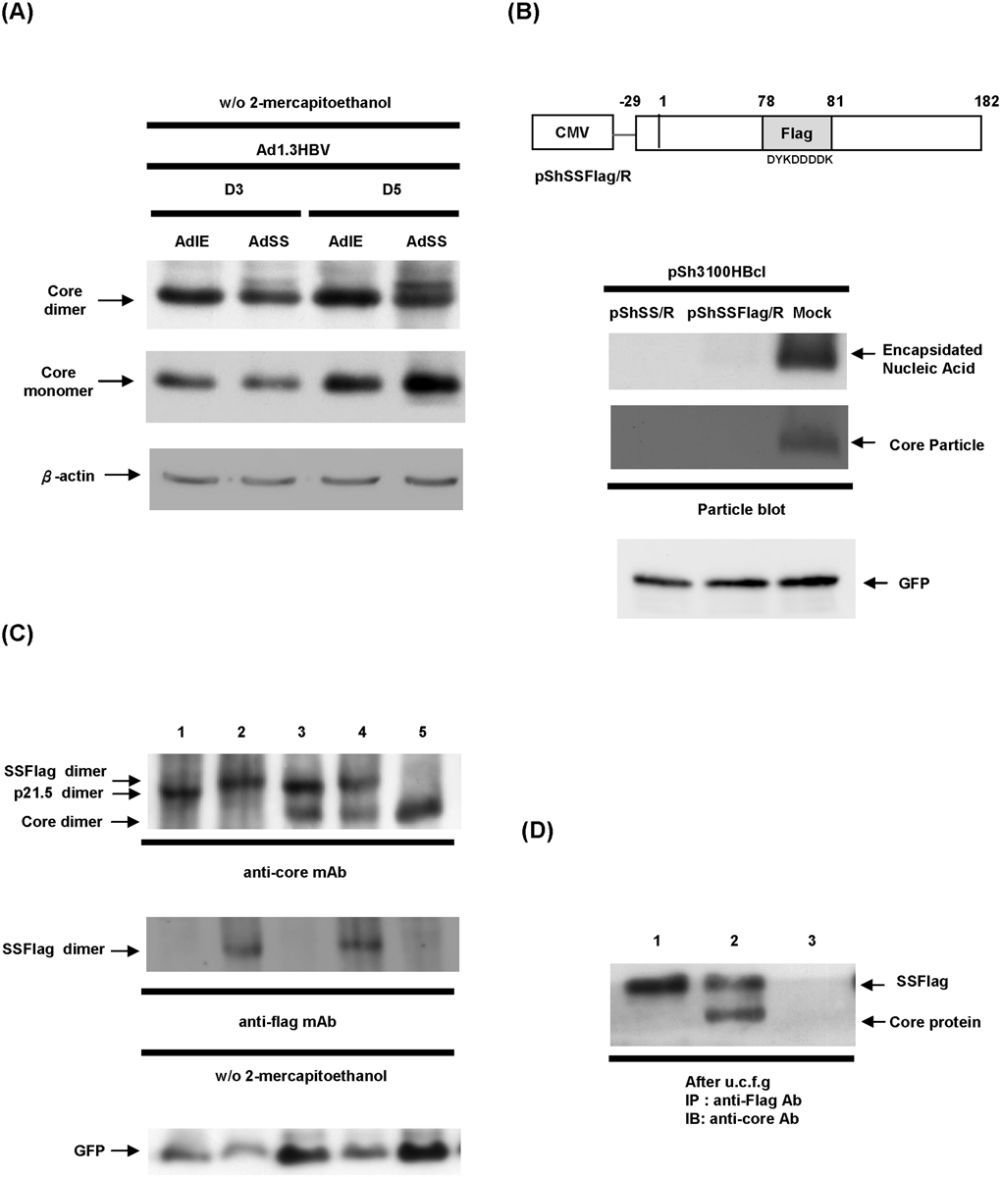
The p21.5 viral protein forms homodimers that assemble with core protein dimers. (A) HepG2 cells were co-transduced with Ad1.3HBV together with either AdSS or AdIE and total cell lysates were harvested at day 3 and day 5 post-transduction. SDS-PAGE was performed on the samples in the absence of 2-mercaptoethanol. The expression levels of core protein monomer and dimer were revealed by western blot analysis using an anti-core antibody. (B) Upper panel, schematic of the coding structure of pShSSFlag/R in which the amino acids 78^th^-81^th^ (DPAS) of the core ORF were replaced by the flag sequence (DYKDDDDK). Lower panel, 293T cells were co-transfected with the indicated plasmids and the expression of intracellular nucleocapsids was determined by particle blot analysis. (C) 293T cells were co-transfected with the indicated plasmids for 3 days, and the cell lysates were harvested and SDS-PAGE was performed without 2-mercaptoethanol. The expression levels of core protein monomer and dimer were examined by western blot analysis using anti-core antibody and anti-FLAG antibody. The GFP signal was used as a loading control. Lane 1: pShSS/R; lane 2: pShSSFlag/R; lane 3: pSh3100HBcl and pShSS/R; lane 4: pSh3100HBcl and pShSSFlag/R; lane 5: pSh3100HBcl and pShuttle/R(Mock). (D) 293T cells were co-transfected with the indicated plasmids for 3 days, and then the cell lysates were harvested and pelleted by sucrose cushion centrifugation. The assembled larger viral components were immnoprecipitated by anti-FLAG antibody, followed by immunoblotting with anti-core antibody. Lane 1: pShSSFlag/R and pShuttle/R; lane 2: pSh3100HBcl and pShSSFlag/R; lane 3: pSh3100HBcl and pShuttle/R.

Since HBV nucleocapsid assembly begins with the dimerization of the core protein, we next examined whether expression of p21.5 interferes with dimerization of the core protein, which would in turn diminish the amount of nucleocapsid produced. To estimate the amount of core protein dimers in the presence of p21.5, cell lysates were harvested from HepG2 cells transduced with the indicated adenoviruses and these samples were separated on SDS-PAGE without adding 2-mercaptoethanol in order to maintain the disulfide-link of the core protein dimer. Western blot analysis showed that overexpression of singly-spliced RNA changed neither the amount of core protein dimer present nor the amount of core protein monomer present (Fig. 3A). To mark p21.5 and to track its distribution during core protein dimerization, an in-frame FLAG epitope (DYKDDDDK) was engineered into the loop (hatched area) of the core coding region of p21.5 in order to generate a p21.5-FLAG fusion protein as described by Scaglioni et al. [18] (Fig. 3B, pShSSFlag/R). On expression, the p21.5-FLAG fusion protein showed a similar inhibitory effect on nucleocapsid formation to that p21.5, which indicates that introduction of a FLAG epitope does not alter the nature of p21.5 (Fig. 3B). To investigate whether the p21.5 and p21.5-FLAG interact with wild-type core protein to form heterodimers, the content of the core protein dimers was separated on SDS-PAGE in the absence of 2-mercaptoethanol and analyzed by immunoblotting with anti-core antibody and anti-FLAG antibody (Fig. 3C). Single bands corresponding to the size of p21.5 dimer and to the size of the p21.5-FLAG protein dimer were detected (Fig. 3C, lane 3 and lane 4) indicating that p21.5 and p21.5-FLAG protein are both able to form homodimers with themselves, but not with the wild-type core protein. However, we cannot fully exclude the possibility that heterodimers may also be formed but at too low a level to be detected. We next investigated whether the p21.5 dimers assemble with the core dimers, and therefore form hybrid assembly components. The larger viral assembly components, but not free viral proteins, were pelleted down through a sucrose cushion by centrifugation as described previously [18]. The content of viral assembly components was then analyzed by immunoprecipitation using anti-FLAG antibody, which was followed by immunoblotting assay using anti-core antibody (Fig. 3D). It was determined that wild-type core protein was co-immnoprecipitated with the p21.5-FLAG protein, which indicates that the wild-type core dimers and the p21.5 homodimers are able to form hybrid assembly components (Fig. 3D, lane 2). Taken together, these results suggest that the p21.5 protein is capable of forming homodimers that are able to interact with core homodimers. This interaction would seem to interfere with regular nucleocapsid formation, which then results in an inhibition of HBV replication.

### The 21.5 kDa viral protein interferes with the formation of intracellular nucleocapsids

**Fig 4:**
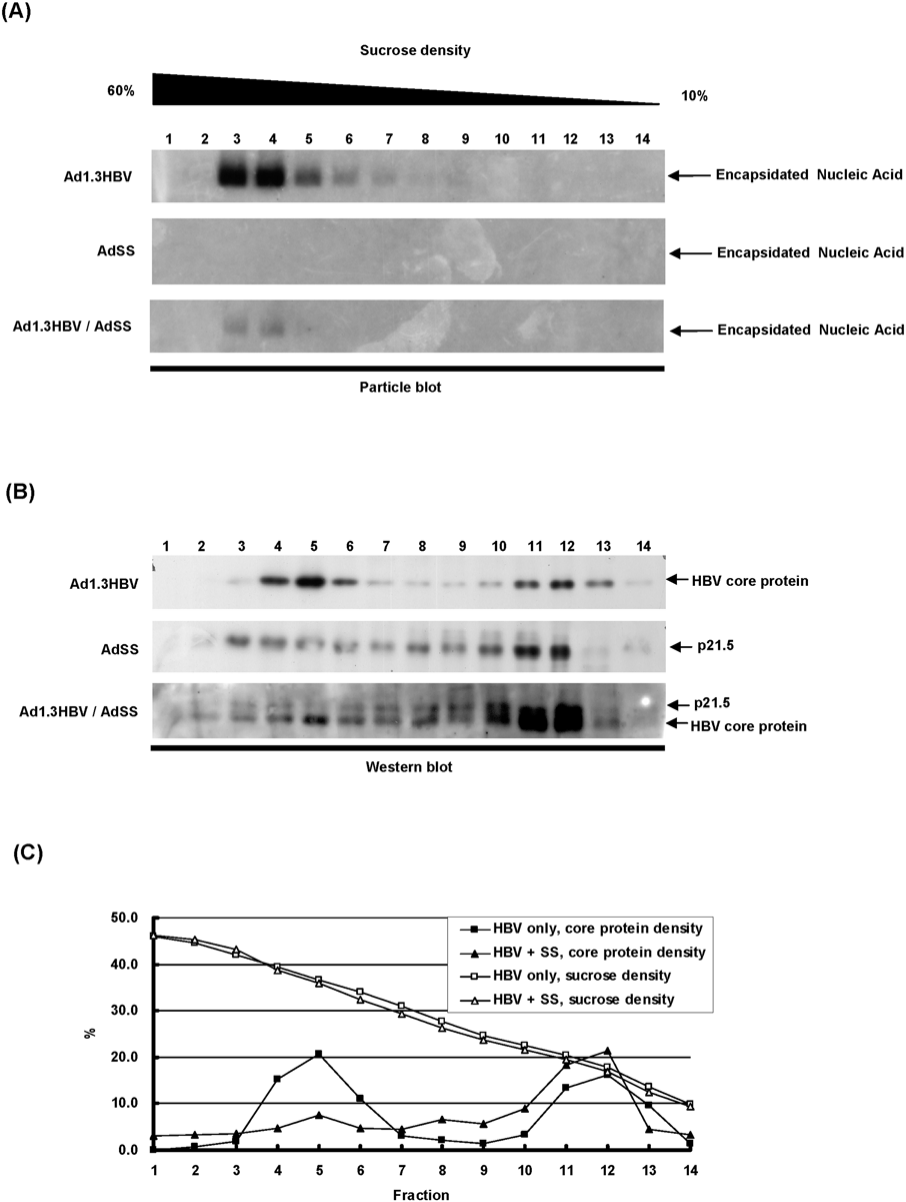
Expression of p21.5 interferes with the process of nucleocapsid formation. HepG2 cells were transduced with Ad1.3HBV and/or AdSS for three days and cell lysates were harvested for sucrose gradient centrifugation (10–60%) and then fractionation was performed. (A) Equal volumes of total protein were separated by electrophoresis on an agarose gel and the expression of nucleocapsid in each fraction was detected by particle blot analysis using an HBx-specific probe. (B) Equal volumes of total protein were separated by electrophoresis on SDS-PAGE and the expression of HBV core protein in each fraction was detected by western blot analysis using an anti-core antibody. (C) The relative proportions of core protein in the fractionated samples (Ad1.3HBV alone or Ad1.3HBV plus AdSS) were estimated and aligned according to the fraction.

Previous studies have reported that intracellular core proteins/particles can be divided into a low-density group (non-particulate core proteins) and a high-density group (capsid particles) after 10%–60% sucrose gradient fractionation; in such circumstances the assembly intermediates are hardly detectable as they exist in very low amounts or for a very short time [21, 22]. To further confirm whether the expression of p21.5 interferes with the process of intracellular nucleocapsid assembly during HBV replication, the populations of intracellular viral particle and genome-free core proteins were separated by sucrose gradient centrifugation and then examined by particle blot and western blot analyses (Fig. 4). The fractionation of the total lysate from Ad1.3HBV-transduced HepG2 cells showed a typical distribution pattern of core proteins, in which the genome-containing viral capsids were located in the high sucrose density fractions (Fig. 4A and 4B, Ad1.3HBV fractions 3–6) and the genome-free core proteins or core dimers were distributed to the low sucrose density fractions (Fig. 4A and 4B, Ad1.3HBV fractions 11–13). On the other hand, when there was expression of the singly-spliced RNA, this resulted in a spread distribution pattern of p21.5 in fractions ranging from low to high sucrose densities (Fig. 4B, AdSS fractions 3–12). Thus, interestingly, the co-expression of singly-spliced RNA and HBV pgRNA dramatically reduced the formation of nucleocapsids as well as altering the distribution pattern of core proteins (Fig. 4A and 4B, Ad1.3HBV/AdSS). The relative proportion of core protein in each fraction was estimated and aligned according to their fractions. Comparative analysis showed that co-expression of p21.5 caused a significant shift in core protein distribution from high-density core species (viral capsids or nucleocapsids) to medium-density and low-density core species (intermediates, core dimers and genome-free core proteins) (Fig. 4C). These findings indicate that the existence of p21.5 is able to interfere with the formation of intracellular nucleocapsids.

### p21.5 protein has properties that are distinct from those of the nonsecreted HBeAg precursor protein p22

**Fig 5:**
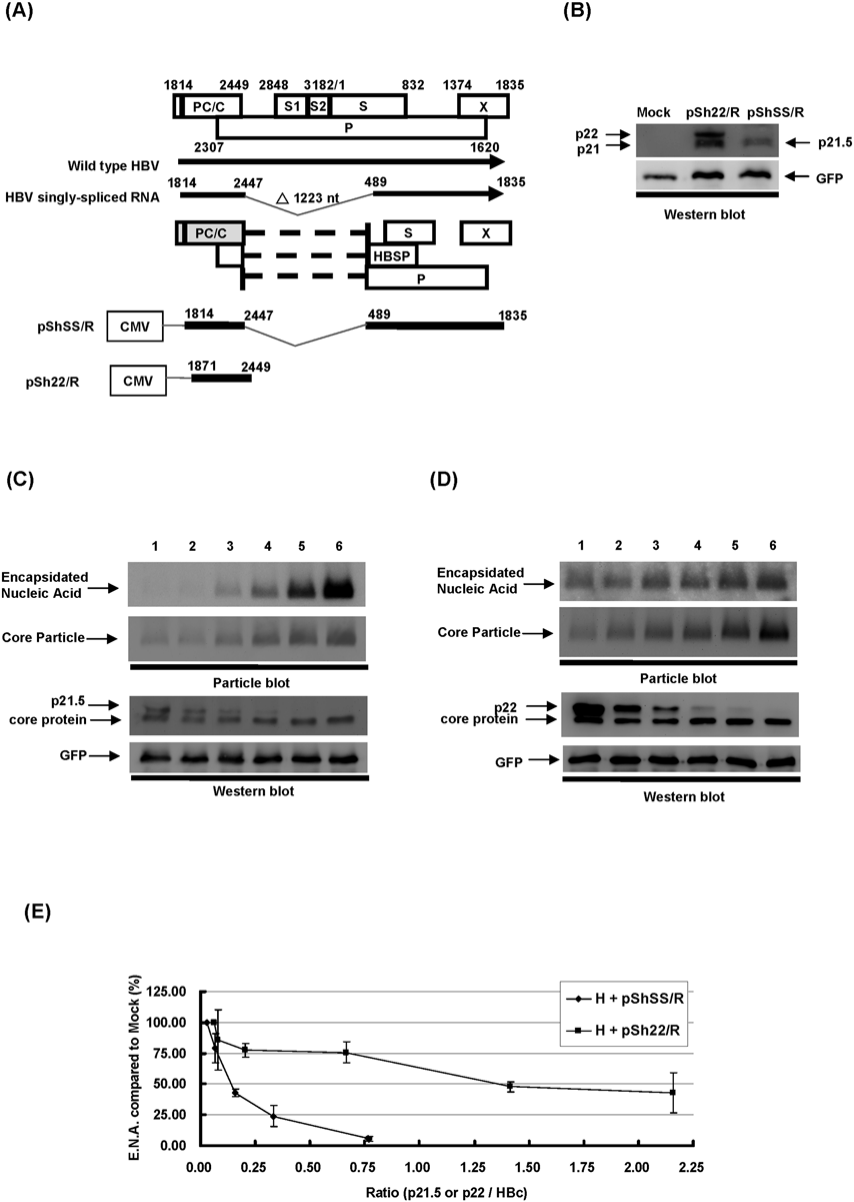
A comparison of p21.5 protein and the nonsecreted HBeAg precursor protein p22. (A) Schematic of the plasmid constructs. pShSS/R, a plasmid expressing full-length singly-spliced RNA. pSh22/R, a plasmid expressing p22 nonsecreted HBeAg precursor protein. (B) Huh7 cells were transfected with pShSS/R, pSh22/R, or control plasmid (Mock). The cells were harvested three days post-transfection for western blot analysis. (C) and (D) Huh7 cells were co-transfected with p1.3HBcl/Hyg together with either pShSS/R or pSh22/R in the ratio of 1:1, 1:0.5, 1:0.25, 1:0.125, 1:0.0625, or with control plasmid in the same ratios (Mock) (lane 1–6). Next, the cells were harvested three days post-transfection and subjected to particle blot and western blot analysis. (E) The relative levels of encapsidated nucleic acid in relation to the ratios of p21.5/wild-type core protein were quantified and compared with those of the p22/wild-type core protein.

It has been reported that p22, a nonsecreted HBeAg precursor protein is also able to exert an inhibitory effect on HBV replication through the formation of hybrid capsids with the HBV core protein [18]. To compare the properties of p21.5 protein to the known p22 protein, Huh7 cells were transfected with plasmids expressing either the p21.5 protein (pShSS/R) or the p22 protein (pSh22/R) (Fig. 5A), and then the migration profiles of the two viral proteins were compared by western blot analysis. As shown in Fig. 5B, pSh22/R plasmid generated two species of viral proteins, the nonsecreted HBeAg precursor (p22) and the wild-type HBc protein (p21), both of which had distinct migration abilities compared to the pShSS/R-encoded p21.5 protein. To investigate the inhibitory efficacies of p21.5 and p22 during the modulation of HBV replication, 1.3HBcl/Hyg together with either pShSS/R or pSh22/R were co-transfected into Huh7 cells at different molecular ratios (from 1:1 to 1:0.0625), and the HBV replication capabilities in relation to the amount of p21.5 or p22 then analyzed by particle blotting and western blotting (Fig. 5C and 5D). It was found that expression of the 2.2 kb singly-spliced RNA is able to efficiently interfere with HBV nucleocapsid formation even when there is very little p21.5 expression (Fig. 5C, lane 4). In contrast, p22 only showed a modest inhibitory effect on HBV replication (Fig. 5D). In order to produce p22 by conversion in its natural way from its precursor protein p25, we constructed pShPC/R and performed an experiment to demonstrate the inhibitory effect of this naturally-produced p22. S2 Fig. showed that pShPC/R exerted similar inhibitory effect on HBV nucleocapsid formation as the construct pSh22/R. Both of pShPC/R and pSh22/R show modest inhibitory effects compared to pShSS/R, the singly-spliced RNA expressing plasmid. The relative levels of encapsidated nucleic acid in relation to the ratios of p21.5 to wild-type core protein were quantified and compared with that of p22 to wild-type core protein (Fig. 5E). In order to obtain an inhibitory effect of 50%, the ratio of p21.5 needed was found to be 0.14 whereas the ratio for p22 at 50% inhibition was 1.36. These findings demonstrate that p21.5 protein is about ten times more potent as an anti-viral molecule than p22 protein in Huh7 cells overexpressing either p21.5 or p22 protein.

### HBV nucleocapsids are inhibited by overexpression of the singly-spliced 2.2 Kb RNA *in vivo*

**Fig 6:**
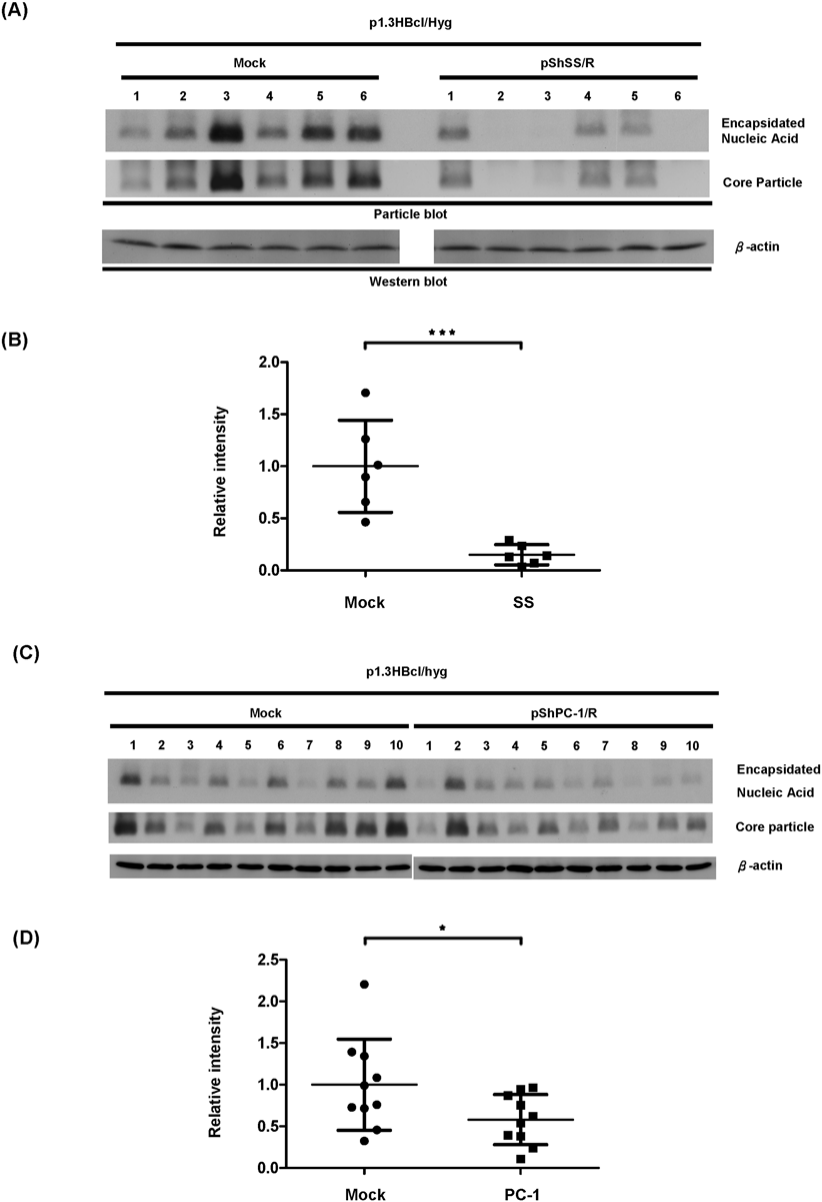
A decrease in the amount of HBV nucleocapsids occurs when there is overexpression of singly-spliced2.2 kb RNA *in vivo*. (A) BALB/c mice were injected with p1.3HBcl/Hyg together with either pShSS/R (N = 6) or p1.3HBcl/Hyg and control plasmid (Mock) (N = 6) by tail vein injection. (C) BALB/c mice were injected with p1.3HBcl/Hyg together with either pShPC-1/R (N = 10) or p1.3HBcl/Hyg and control plasmid (Mock) (N = 10) by tail vein injection. The mice were sacrificed three days post-injection and total cell lysate from the liver was then harvested in order to determine the amount of intracellular nucleocapsid by particle blot analysis. The expression of β-actin was used as a loading control. (B) and (D) The signal intensities of the encapsidated nucleic acid were evaluated and normalized with serum HBsAg respectively, and the results are shown as scatter plot. The results were analyzed by unpaired Student’s t-test (***P<0.001, *P<0.05).

Based on the fact that our findings indicate that the singly-spliced RNA is able to interfere with the formation of intracellular nucleocapsids via the p21.5 protein *in vitro*, we next explored whether singly-spliced RNA is able to exert its inhibitory effect *in vivo*. An HBV-expressing plasmid (p1.3HBcl/hyg), together with either singly-spliced RNA-expressing plasmid (pShSS/R) or mock control plasmid (pShuttle/R), were co-injected into the tail vein of BALB/c mice as described by Huang et al. [23]. As shown in Fig. 6A, the amounts of intracellular nucleocapsids isolated from the mice that had been treated with the singly-spliced RNA were significantly lower than that of the control mice. A scatter plot of the signal intensities for the encapsidated nucleic acid from the two sets of mice is shown (Fig. 6B). The expression level of serum HBsAg in each mouse receiving hydrodynamic injection was shown in S3A Fig. As a result of the fact that there are multiple protein species encoded by the singly-spliced RNA, in order to demonstrate whether the inhibitory effect on capsid formation is a result of the p21.5 protein encoded by singly-spliced RNA, we used a p21.5 expressing plasmid (pShPC-1/R) rather than pShSS/R for co-injection with HBV-expressing plasmid (p1.3HBcl/hyg) into the tail vein of the BALB/c mice. The findings in Fig. 6C show that the amount of intracellular nucleocapsid isolated from the mice expressing p21.5 was significantly lower than the amount isolated from the control mice. A scatter plot of the signal intensities of the encapsidated nucleic acid from the two sets of mice is shown in Fig. 6D. The level of HBsAg in mouse serum was shown in S3B Fig. Taking these findings in combination, our current investigation has demonstrated that the p21.5 protein, which is encoded by the 2.2 kb singly-spliced RNA, plays an inhibitory role in the modulation of HBV replication both *in vitro* and *in vivo*.

## Discussion

A variety of viruses generate spliced viral RNAs or splice-related proteins that act as functional molecules and are able to modulate host-virus interactions [24–28]. Retroviruses and pararetroviruses generate functional spliced viral products that regulate viral replication [27, 28]. Two important human immunodeficiency virus proteins, tat and rev, are both generated by alternative splicing and have been reported to transactivate viral/cellular genes and to regulate RNA splicing, respectively [27]. Human T cell leukemia virus type 1 generates a novel spliced RNA that encodes p21X; this has been suggested to play a crucial role in regulating viral replication [28]. In addition to these RNA viruses, BK virus, a member of the polyomavirus family, generates a truncated form of the viral protein truncTAg by alternative splicing. This protein has been found to localize to the nucleus and is likely to be relevant to transformation [24]. Human cytomegalovirus generates two spliced viral RNAs, UL128 and UL131A, and these have been suggested to be deleterious to its growth in cell culture [25]. Kaposi’s sarcoma-associated herpes virus expresses a family of splice-generated membrane proteins that interact with host TRAFs and are likely to be involved in the modulation of virus latency in the same way as the LMP2A and LMP1 proteins of Epstein-Barr virus [26]. All of these independent lines of evidence suggest that viruses are able to generate viral RNAs or viral proteins by alternative splicing that are involved in regulating the viral life cycle during virus infection.

**Fig 7:**
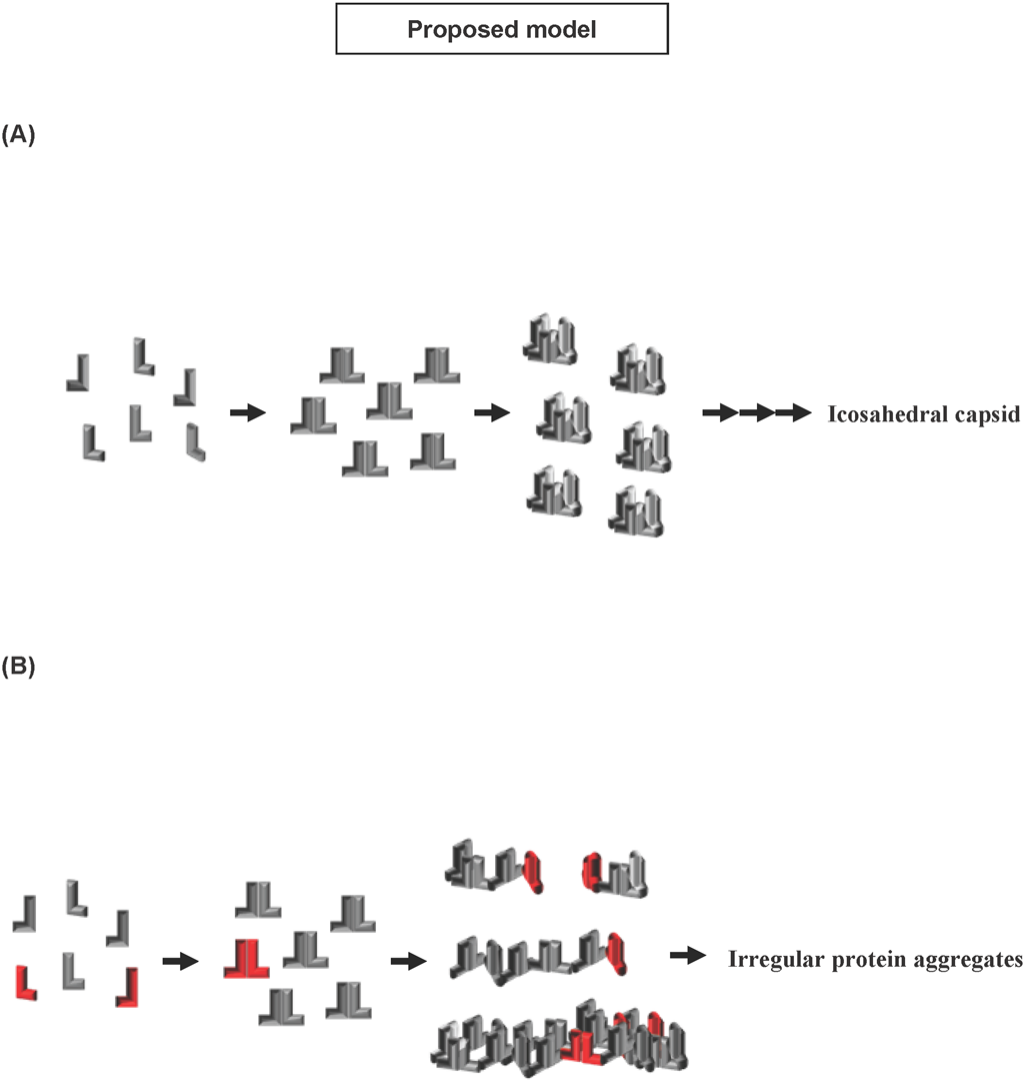
A model outlining the inhibitory effect of the p21.5 protein encoded by singly-spliced RNA on HBV replication. (A) During regular HBV nucleocapsid assembly, viral core monomers initially form core homodimers and these then further assemble into the hexamer intermediates before the quick formation of the icosahedral nucleocapsid. (B) In the presence of p21.5, p21.5 proteins initially interact with each other and form homodimers. These p21.5 homodimers and the core homodimers then form mosaic viral particles that interfere with the formation of appropriate icosahedral nucleocapsid structure. The wild-type core protein is shown in gray; the p21.5 protein, which is encoded from singly-spliced RNA, is shown in red.

The existence of HBV spliced RNAs has been known for decades. Considerable effort has been put into elucidating their biological functions during HBV replication. Increasing evidence suggests that HBV spliced RNAs are functional molecules that play diverse biological roles during viral infection such as being critical factors for viral replication [29], forming defective viral particles during persistent infection [8, 9], being involved in hepatopathogenesis [13–16], encoding a pluritropic transcription activator [30], encoding a viral structural protein [31], and encoding a modulator for HBV replication [32]. Taken together, these studies suggest that spliced HBV RNAs and the encoded viral proteins have probably evolved as a means of regulating viral replication, persistence, and/or pathogenesis during HBV infection. In the present study we provide evidence that singly-spliced RNA may play an inhibitory role in the modulation of HBV replication. Overexpression of singly-spliced RNA does slightly inhibit the synthesis of pgRNA; nevertheless, this RNA primarily interferes with viral particle formation (Fig. 1A and 1B). This finding is supported by the fact that overexpression of singly-spliced RNA is able to inhibit the self-assembly of core particles even in the absence of pgRNA (Fig. 1C). A nonsense mutation (pShSScore^-^/R) or a precore ATG mutation (pShSS_PCmut/R) of the singly-spliced RNA was found to completely abolish its inhibitory activity, which indicates that a viral protein derived from the precore protein is responsible for the inhibition of virus replication that is mediated by the singly-spliced RNA (Fig. 2). The expression of p21.5 was found to decrease the level of nucleocapsids. This interference could be achieved with a very low amount of p21.5, as little as 5% of that of the core protein or even less (Fig. 5C, lane 4). Further studies showed that this p21.5 protein is able to form homodimers (Fig. 3), but fails to self-assemble into replication competent particles. This result is consistent with previous reports wherein the ectopic expression of singly-spliced RNA alone was unable to form core particles in transient transfection experiments [7, 9]. It is very likely that the p21.5 homodimers and core dimers assemble into mosaic viral particles and that this event prevent the formation of regular nucleocapsids. Moreover, the overexpression of singly-spliced RNA in the mouse hepatocytes significantly interfered with the formation of intracellular nucleocapsids *in vivo* (Fig. 6 A). We further demonstrated that the effect of singly-spliced RNA on nucleocapsid formation is due to the expression of p21.5. This was done using pShPC-1/R, which only expresses p21.5 protein and removes from the equation other possible proteins encoded by the singly-spliced RNA *in vivo* (Fig. 6C). The difference between Fig. 6A and 6C might be the result of a possible effect of the 3'UTR of the singly-spliced RNA on mRNA posttranscriptional regulation. It has been suggested that transcripts covering the HBV X coding region are involved in the HBV mRNA stability [33–35]. The 3'UTR of the singly-spliced RNA is absent in the construct pShPC-1/R and this might have produced the difference between Fig. 6A and 6C. Taking the above findings together, a model outlining the inhibitory effect of the p21.5 protein encoded by the singly-spliced RNA on the modulation of HBV replication is presented in Fig. 7.

Although the exact coding sequence of the p21.5 protein identified in this study is not yet completely clarified, our findings confirm that p21.5 is encoded from the precore region of the singly-spliced RNA and a high level of secreted HBeAg can be detected in the culture medium of singly-spliced RNA-expressing cells. Peptide mapping by LC-MS/MS has indicated that the N-terminus sequence of p21.5 seems to be identical to that of p22 (data not shown). Therefore, the predicted coding sequence of p21.5 is that of the intracellular HBeAg precursor, but being one-amino acid (Cys183) shorter than that of previously identified HBeAg precursor p22 [7]. As shown in S4 Fig., pShSS_prestop mutant/R generated a shortened viral protein with prestop codon at the 7^th^ amino acid upstream of the authentic stop codon of p21.5, which showed p21.5-like inhibitory effect and merged along with p21 on the western blot. It is well known that the C-terminus of the precore protein, including the residue Cys183, is involved in the protein folding that influences C-terminal cleavage during HBe maturation [21, 36, 37]. As a consequence, the one-amino acid (Cys183) difference between p21.5 and p22 may result in a very distinct protein folding structure and thus different biological and biochemical properties, namely the ability to interfere with HBV nucleocapsid formation. Indeed, our findings indicate that p21.5 is about ten times more efficient in terms of destabilizing HBV nucleocapsid than p22 when either the p21.5 protein or the p22 protein is overexpressed in Huh7 cells (Fig. 5E). To further compare the difference between p21.5 and p22, we performed sucrose gradient fractionation to demonstrate the pattern of intracellular core species when 293T cells were transiently transfected with pShSS/R or pSh22/R alone, or in combination with p1XHBV. The sucrose density of each fraction was determined and shown in S5A Fig. The fractionated samples were then separated by SDS-PAGE, and the distribution patterns of the core species were determined by immunoblotting using an anti-core antibody. The fractionation of the total lysate from 293T cells transfected with p1XHBV showed a typical distribution pattern of core proteins subgrouped into two groups (the first panel in S5B Fig.). The fractionation of pShSS/R showed a spread distribution pattern of p21.5 in fractions ranging from low to high sucrose densities (the second panel in S5B Fig.); whereas the fractionation of pSh22/R showed that p22 proteins were mainly located in the high-density fractions (the third panel in S5B Fig.). Furthermore, intracellular core species showed a spread distribution pattern across the low-density fractions when the cells co-expressing HBV and p21.5, indicating that p21.5 may disrupt the HBV nucleocapsid formation. By contrast, the distributions of core species in cells co-expressing HBV and p22 protein were located primarily in the high-density fractions (the fourth and fifth panels in S5B Fig.). These findings indicate that p21.5 and p22 would assemble into distinct core species inside HBV-expressing cells. Taken together, all lines of evidence suggested that p22 and p21.5 are not the same viral protein since they show distinct biochemical properties, such as different migration mobility, inequivalent inhibition capability, and forming distinct intracellular core species. Our findings also suggest the C-terminal Cys183 is critical to the formation of HBV viral particle. This suggestion is supported by a previous study showing that the Cys183 is a crucial residue to the formation of disulfide bonds during dimer-dimer interaction; these bonds stabilize the icosahedral structure of the viral capsid [38]. When the Cys183 residue is missing, the p21.5 homodimers embedded in the mosaic viral particles fail to be able to maintain the regular icosahedral structure and this may then creates an inhibitory effect on HBV nucleocapsid assembly.

Although singly-spliced RNA has been known for over two decades and has been well characterized [7–9], the existence of the p21.5 viral protein encoded by singly-spliced RNA has not been identified until now. Failure to detect p21.5 in previous studies is very likely due to its low expression level and its high similarity to the wild-type core protein. It has been reported that the precore and pregenomic promoters are genetically distinct and differentially regulated [39]. Since the precore promoter is negatively regulated by liver-enriched transcription factors such as HNF4 and TR2 [39, 40], the transcription machinery favors transcription initiation from the pregenomic promoter, rather than the precore promoter. As a consequence, it is no surprise that expression of the p21.5 protein is hardly detectable during HBV replication in a cell culture system. However, this may not be the case in patients with acute hepatitis B infection. A previous study has suggested that high levels of HBV genome expression might titrate out the amounts of negatively-regulative transcription factors that bind to the precore promoter, thereby leading to the activation of the precore promoter and an enhancement of its transcription [40]. In this manuscript, our findings provide a possible regulatory role for the singly-spliced RNA in that an elevation of its expression might feedback and regulate/modulate HBV replication during establishment of HBV infection. This hypothesis is supported by the detection of higher levels of singly-spliced RNA in acute hepatitis patients who progress to chronic hepatitis [9] and by the higher frequency of the detection of spliced defective viral DNA in patients with a high viral load, namely >10^4^ IU/ml [41].

Our current study suggests that expression of minute amounts of p21.5 is sufficient to interfere with nucleocapsid formation, raising the possibility that the elevation of singly-spliced RNA in CHB patients may play a functional role in the feedback modulation of HBV replication. In this case, the increased level of p21.5 translated from the singly-spliced RNA would seem to diminish the formation of nucleocapsid and thus contribute to the establishment of a viral persistent infection. Evolving such an inhibitory effect involving destabilization or disruption of viral nucleocapsids constitutes a common anti-viral strategy for controlling of HBV infection. For example, some antiviral cytokines, such as tumor necrosis factor alpha and various interferons, have been shown to destabilize pre-existing viral particles through unknown mechanisms [19, 42, 43]. The antiviral compounds, the heteroarylpyrimidines (HAP), were found to prevent capsid maturation by interacting with a region of the core protein known to be critical for dimerization, multimerization, and viral capsid formation [44]. Our study has shown that the p21.5 protein, which is encoded by the 2.2kb singly-spliced RNA, is able to efficiently decrease the amount of HBV nucleocapsids present both *in vitro* and *in vivo*. This may provide insights into the development of potential therapeutic strategies for controlling HBV replication.

## Supporting Information

S1 Fig The expression level of core mRNA in the present of HBV singly-spliced RNA. Huh7 cells were co-transfected with pShCore/R together with either pShSS/R or a control plasmid (Mock), and total mRNA was harvested at day 3 post-transfection. The expression of core mRNA was confirmed by northern blot analysis via hybridization with an HBc-specific probe. The expression level of co-transfected GFP was used as a transfection control and 18s rRNA was used as a loading control.
(TIFF)

S2 Fig The dosage effect of pShPC/R in modulation of HBV encapsidation. (A) Schematic of the products of the HBV precore/core gene. pShPC/R was constructed by cloning the coding sequence of the HBV precore protein into the pShuttle/R vector. (B) Huh7 cells were co-transfected with p1.3HBcl/Hyg and pShPC/R in the ratio of 1:1, 1:0.5, 1:0.25, 1:0.125, 1:0.0625, or control plasmid (Mock) (lane 1–6) and the cells were harvested three days post-transfection for particle blot and western blot analyses. Top two panels, the expression levels of nucleocapsid were analyzed by particle blot analysis. The encapsidated nucleic acid was revealed by hybridizing with an HBx-specific probe. Lower two panels, the expression levels of HBV core protein and GFP protein were examined by western blot analysis.
(TIFF)

S3 Fig The levels of HBsAg in mice sera detected by ELISA. (A) BALB/c mice were injected with p1.3HBcl/Hyg together with either pShSS/R (N = 6) or p1.3HBcl/Hyg and control plasmid (Mock) (N = 6) by tail vein injection. (B) BALB/c mice were injected with p1.3HBcl/Hyg together with either pShPC-1/R (N = 10) or p1.3HBcl/Hyg and control plasmid (Mock) (N = 10) by tail vein injection. The mice were sacrificed three days post-injection and the sera were harvested to determine the amount of secreted HBsAg. The cutoff values of the ELISA assay in S3A Fig. and S3B Fig. are 6.81 ng/ml and 5.62 ng/ml, respectively.
(TIFF)

S4 Fig A shortened p21.5 encoded by pShSS_prestop mutant/R shows an inhibitory effect on HBV encapsidation. (A) Top panel, a schematic of the structure and open reading frames of the singly-spliced RNA. Lower panel, a schematic of the plasmid constructs. pShSS/R, a plasmid expressing full-length singly-spliced RNA. pShSS_prestop mutant/R, a plasmid expressing singly-spliced RNA with a prestop mutation at the position 2428 (numbering starting at the EcoRI site) within the precore/core ORF. (B) Huh7 cells were transfected with the indicated plasmids. Lane 1: p1.3HBcl/Hyg and pShSS/R; lane 2: p1.3HBcl/Hyg and pShSS_prestop mutant/R; lane 3: p1.3HBcl/Hyg and control plasmid (Mock). The cells were harvested three days post-transfection for particle blot and western blot analysis. Top two panels, the expression levels of nucleocapsid were analyzed by particle blot analysis. The encapsidated nucleic acid was revealed by hybridizing with an HBx-specific probe. Lower two panels, the expression levels of HBV core protein and GFP protein were examined by western blot analysis.
(TIFF)

S5 Fig Different core protein distribution patterns of wild-type core, p21.5 and p22 protein in sucrose gradient fractionation. 293T cells were transiently transfected with p1XHBV, pShSS/R or pSh22/R alone, or p1XHBV combined with pShSS/R or pSh22/R for three days and cell lysates were harvested for sucrose gradient centrifugation (10–60%). (A) The sucrose densities of fractioned samples were detected and shown. (B) Distribution patterns of core species after sucrose gradient fractionation were assessed by western blot analysis. Equal volumes of total protein were separated by electrophoresis on SDS-PAGE, and the distribution of core proteins species in each fraction was detected by western blot analysis using an anti-core antibody. The mobility of wild type core, p21.5 and p22 was not clearly resolved in the blot.
(TIFF)
