# Supplementary figures and images for: The Inhibitory Effect of the Hepatitis B Virus Singly-Spliced RNA-Encoded p21.5 Protein on HBV Nucleocapsid Formation
Source: PLoS One. 2015 Mar 18;10(3):e0119625. doi: 10.1371/journal.pone.0119625 (PMC4364729; doi:10.1371/journal.pone.0119625)

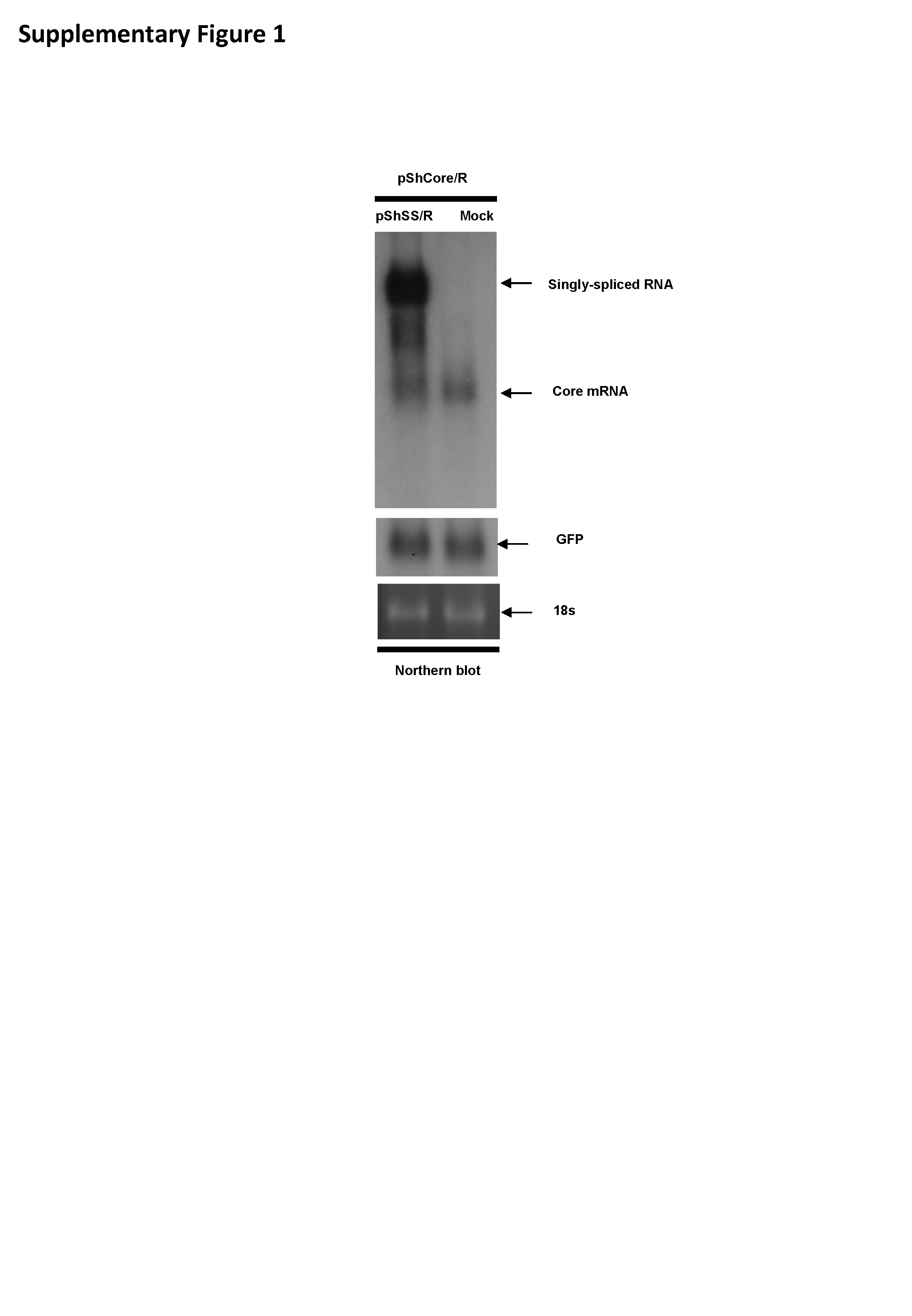

Supplement: S1 Fig — Huh7 cells were co-transfected with pShCore/R together with either pShSS/R or a control plasmid (Mock), and total mRNA was harvested at day 3 post-transfection. The expression of core mRNA was confirmed by northern blot analysis via hybridization with an HBc-specific probe. The expression level of co-transfected GFP was used as a transfection control and 18s rRNA was used as a loading control. (TIFF) [file pone.0119625.s001.tiff]

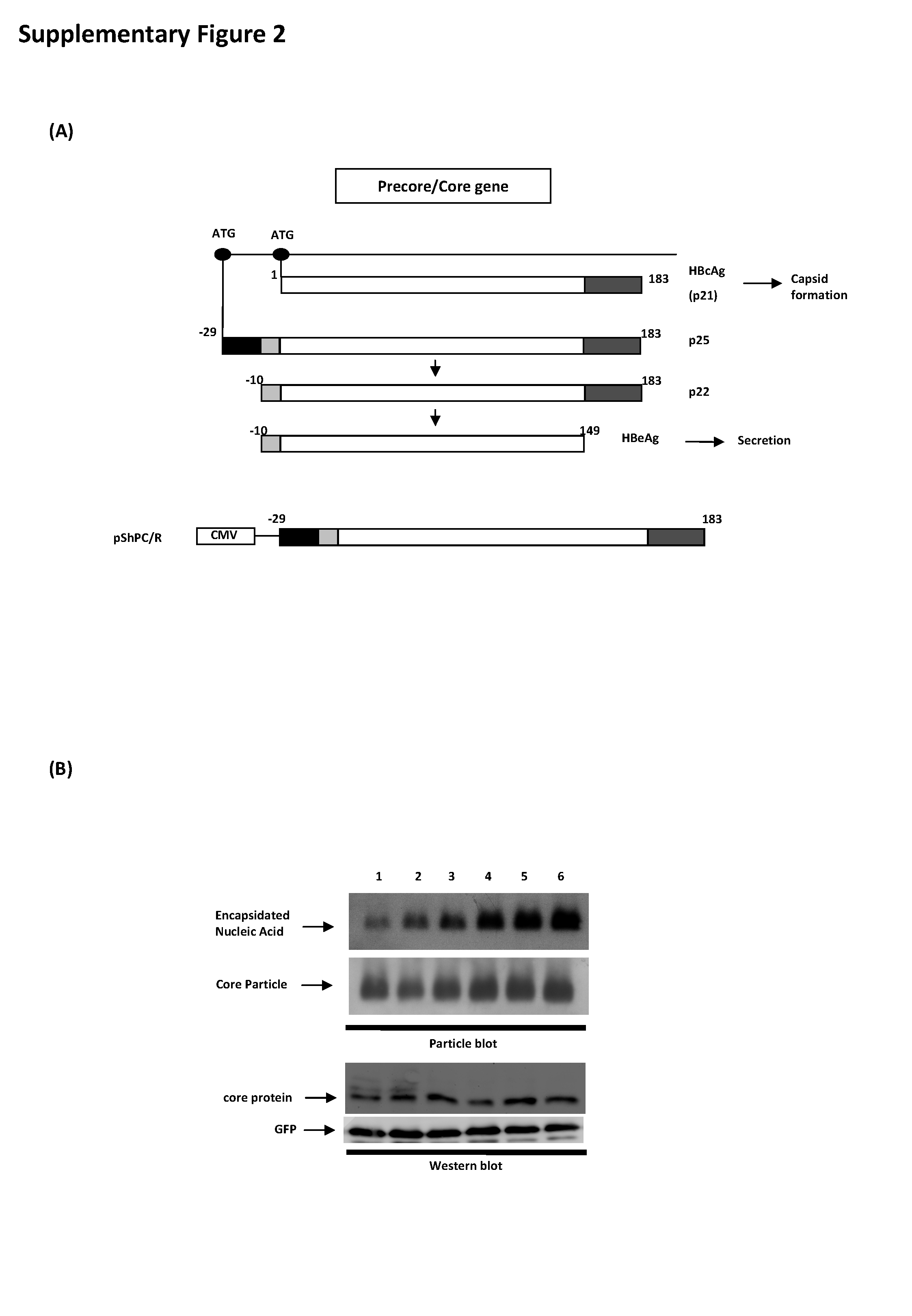

Supplement: S2 Fig — (A) Schematic of the products of the HBV precore/core gene. pShPC/R was constructed by cloning the coding sequence of the HBV precore protein into the pShuttle/R vector. (B) Huh7 cells were co-transfected with p1.3HBcl/Hyg and pShPC/R in the ratio of 1:1, 1:0.5, 1:0.25, 1:0.125, 1:0.0625, or control plasmid (Mock) (lane 1–6) and the cells were harvested three days post-transfection for particle blot and western blot analyses. Top two panels, the expression levels of nucleocapsid were analyzed by particle blot analysis. The encapsidated nucleic acid was revealed by hybridizing with an HBx-specific probe. Lower two panels, the expression levels of HBV core protein and GFP protein were examined by western blot analysis. (TIFF) [file pone.0119625.s002.tiff]

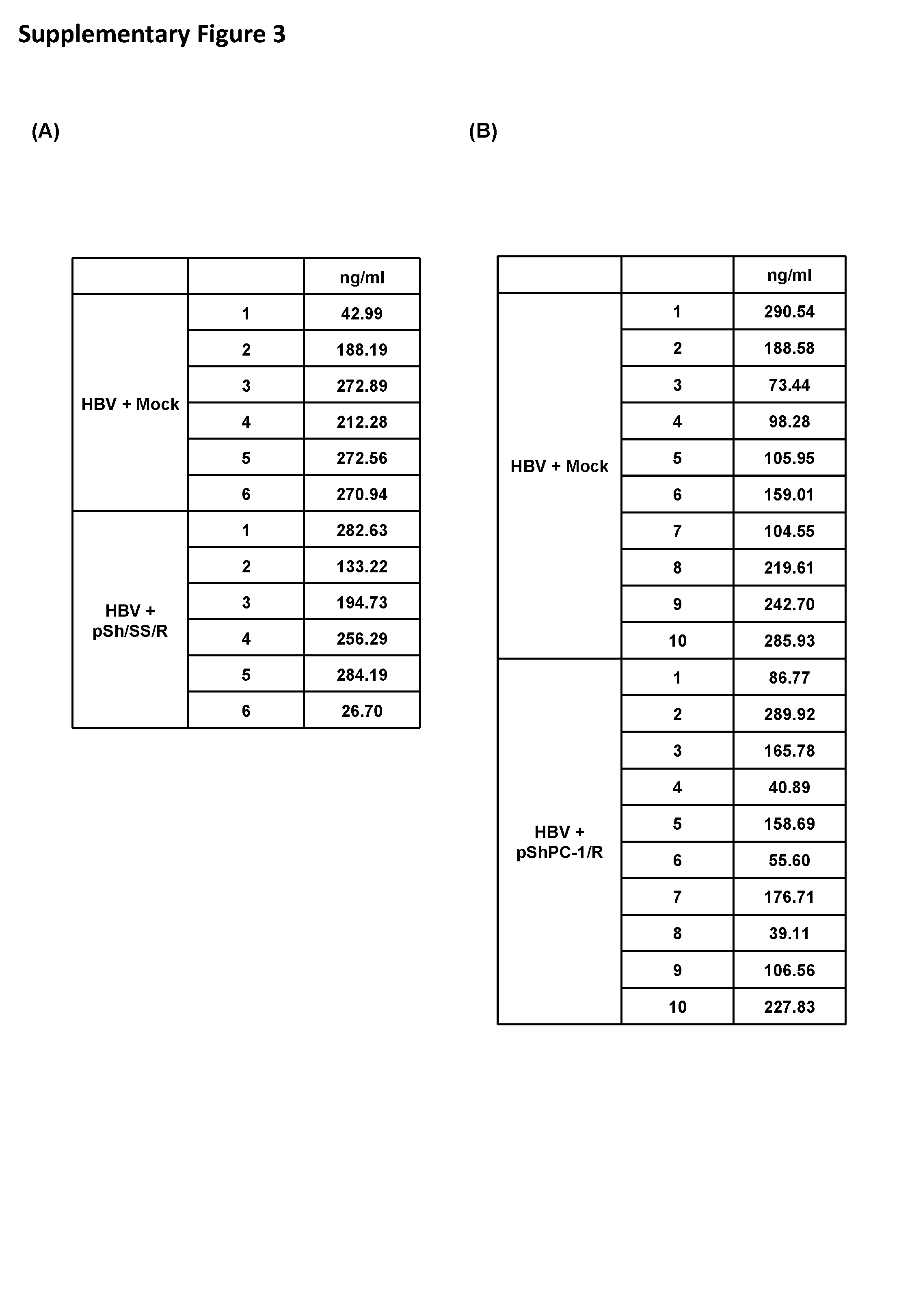

Supplement: S3 Fig — (A) BALB/c mice were injected with p1.3HBcl/Hyg together with either pShSS/R (N = 6) or p1.3HBcl/Hyg and control plasmid (Mock) (N = 6) by tail vein injection. (B) BALB/c mice were injected with p1.3HBcl/Hyg together with either pShPC-1/R (N = 10) or p1.3HBcl/Hyg and control plasmid (Mock) (N = 10) by tail vein injection. The mice were sacrificed three days post-injection and the sera were harvested to determine the amount of secreted HBsAg. The cutoff values of the ELISA assay in S3A Fig. and S3B Fig. are 6.81 ng/ml and 5.62 ng/ml, respectively. (TIFF) [file pone.0119625.s003.tiff]

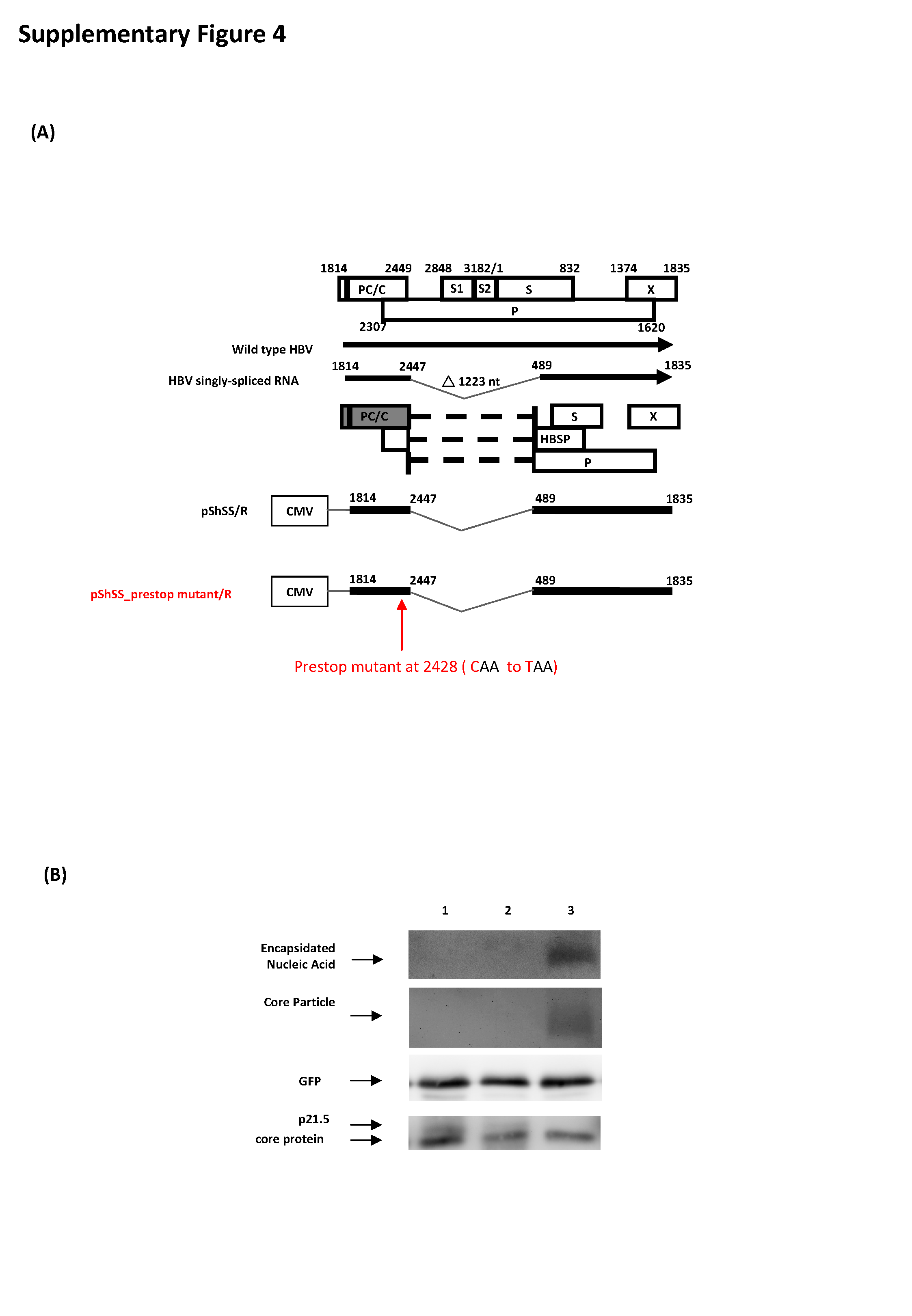

Supplement: S4 Fig — (A) Top panel, a schematic of the structure and open reading frames of the singly-spliced RNA. Lower panel, a schematic of the plasmid constructs. pShSS/R, a plasmid expressing full-length singly-spliced RNA. pShSS_prestop mutant/R, a plasmid expressing singly-spliced RNA with a prestop mutation at the position 2428 (numbering starting at the EcoRI site) within the precore/core ORF. (B) Huh7 cells were transfected with the indicated plasmids. Lane 1: p1.3HBcl/Hyg and pShSS/R; lane 2: p1.3HBcl/Hyg and pShSS_prestop mutant/R; lane 3: p1.3HBcl/Hyg and control plasmid (Mock). The cells were harvested three days post-transfection for particle blot and western blot analysis. Top two panels, the expression levels of nucleocapsid were analyzed by particle blot analysis. The encapsidated nucleic acid was revealed by hybridizing with an HBx-specific probe. Lower two panels, the expression levels of HBV core protein and GFP protein were examined by western blot analysis. (TIFF) [file pone.0119625.s004.tiff]

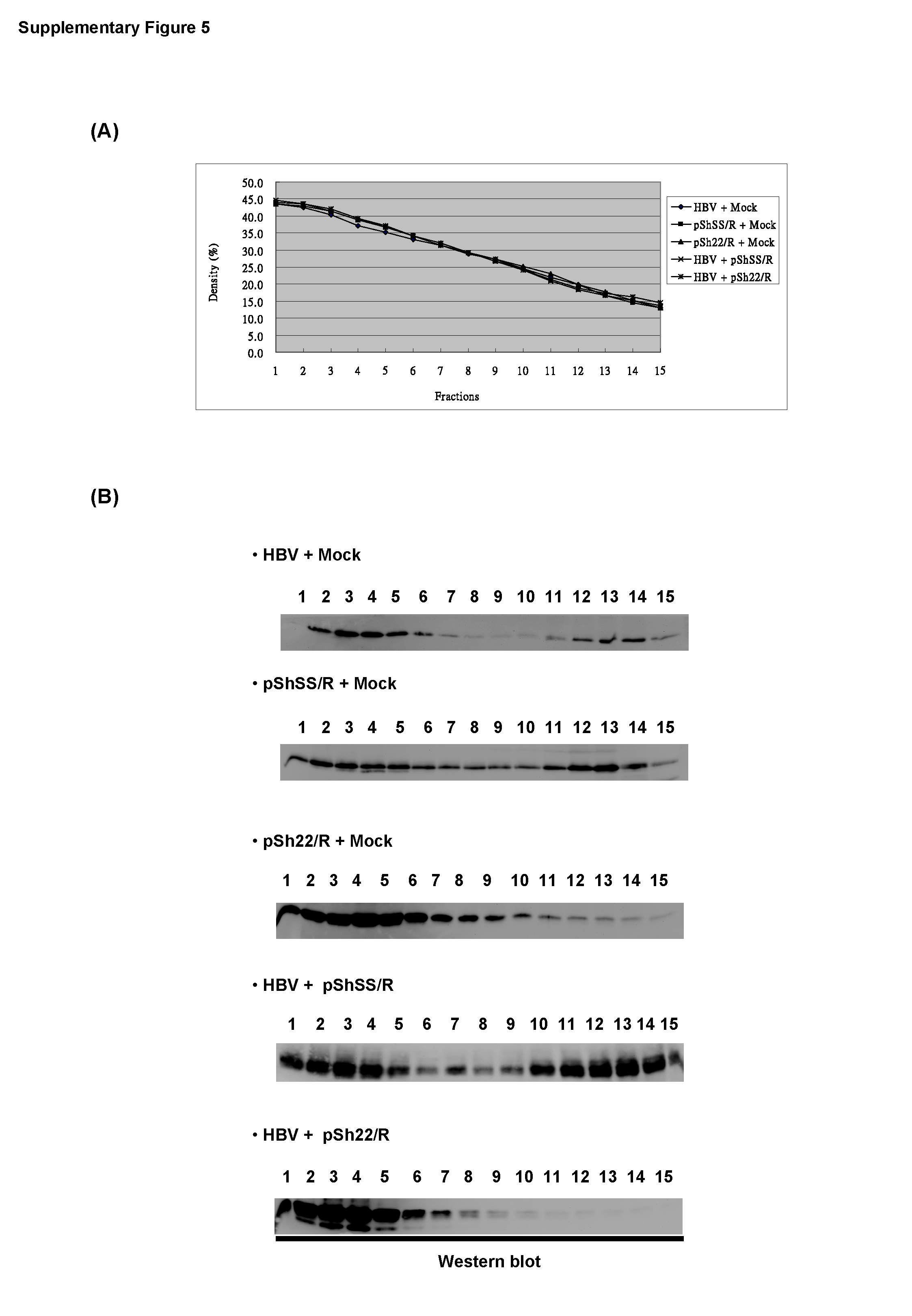

Supplement: S5 Fig — 293T cells were transiently transfected with p1XHBV, pShSS/R or pSh22/R alone, or p1XHBV combined with pShSS/R or pSh22/R for three days and cell lysates were harvested for sucrose gradient centrifugation (10–60%). (A) The sucrose densities of fractioned samples were detected and shown. (B) Distribution patterns of core species after sucrose gradient fractionation were assessed by western blot analysis. Equal volumes of total protein were separated by electrophoresis on SDS-PAGE, and the distribution of core proteins species in each fraction was detected by western blot analysis using an anti-core antibody. The mobility of wild type core, p21.5 and p22 was not clearly resolved in the blot. (TIFF) [file pone.0119625.s005.tiff]
